# The Aversive Effect of Electromagnetic Radiation on Foraging Bats—A Possible Means of Discouraging Bats from Approaching Wind Turbines

**DOI:** 10.1371/journal.pone.0006246

**Published:** 2009-07-16

**Authors:** Barry Nicholls, Paul A. Racey

**Affiliations:** School of Biological Sciences, University of Aberdeen, Aberdeen, United Kingdom; University of Bern, Switzerland

## Abstract

Large numbers of bats are killed by collisions with wind turbines and there is at present no accepted method of reducing or preventing this mortality. Following our demonstration that bat activity is reduced in the vicinity of large air traffic control and weather radars, we tested the hypothesis that an electromagnetic signal from a small portable radar can act as a deterrent to foraging bats. From June to September 2007 bat activity was compared at 20 foraging sites in northeast Scotland during experimental trials (radar switched on) and control trials (no radar signal). Starting 45 minutes after sunset, bat activity was recorded for a period of 30 minutes during each trial and the order of trials were alternated between nights. From July to September 2008 aerial insects at 16 of these sites were sampled using two miniature light-suction traps. At each site one of the traps was exposed to a radar signal and the other functioned as a control. Bat activity and foraging effort per unit time were significantly reduced during experimental trials when the radar antenna was fixed to produce a unidirectional signal therefore maximising exposure of foraging bats to the radar beam. However, although bat activity was significantly reduced during such trials, the radar had no significant effect on the abundance of insects captured by the traps.

## Introduction

The UK government is committed to ensure that 10% of the country's electricity will be generated from renewable sources by 2010 with an aspiration to double this figure by 2020. Unfortunately the drive to ameliorate the indirect impact of energy production on the environment has led to a more immediate impact on local fauna. The exploitation of wind as a renewable and pollution-free source of energy has led to the proliferation of wind farms across the UK where 206 are currently operational, comprising 2381 turbines and with an estimated 444 sites proposed for future development [1]. Several studies have highlighted the problem of birds colliding with turbines placed along traditional migratory routes [2]–[6] but until recently the impact of wind turbines on bats has received little attention.

The scale of the problem became apparent in 2004 when, during a six-week period, an estimated 1,764 and 2,900 bat fatalities were recorded at two wind farms in Pennsylvania and West Virginia respectively [7]. The number of collision mortalities reported in America are greater than in Europe, where surveys have begun more recently. However, 15 of the 35 species of European bat have been recorded as regular victims of turbine collisions, and an Intersessional Working Group of Eurobats listed 20 species thought to be at risk of collision due to their foraging and commuting behaviour [8]. Currently, research in Europe is concentrated on arriving at scientifically credible mortality estimates to assess the extent of the problem. Although this is clearly important, the rapid proliferation of wind turbines requires a more urgent response. Research has to be focussed on the underlying reasons behind these collisions and potential methods of mitigation to prevent what is undoubtedly an increasing threat to bat populations.

Attempts at mitigating bird collisions with wind turbines have typically involved the application of visual stimuli to increase the conspicuousness of the turbine blades [9], [10], but for bats, where audition is the primary sensory modality, this is clearly not appropriate. The design of an acoustic deterrent for bats, as used to mitigate cetacean entanglement in drift nets [11]–[13], is complicated by the intrinsic properties of ultrasound, which attenuates rapidly in air [14]. Despite this inherent problem, a recent study [15] revealed a significant aversive response by big brown bats (*Eptesicus fuscus*) following exposure to broadband white noise in a laboratory. However, when an acoustic deterrent was deployed at a wind farm in New York State, USA, results were more equivocal, and researchers concluded that the acoustic envelope of the deterrent system was probably not large enough to consistently deter the activity of bats within the large volume of the rotor-swept zone [16].

A more promising solution is offered by curtailing the operations of wind turbines during high-risk periods. A substantial portion of bat fatalities at operating wind farms occurs during relatively low-wind conditions during the bat migration period [17]. Some curtailment of turbine operations during these conditions, and during this period, has been proposed as a possible means of reducing impacts to bats [17], [18]. Recent results from studies in Canada [19] and North America [20] indicate that changing turbine “cut-in speed” (i.e., the wind speed at which wind generated electricity enters the power grid) from the customary 3.5–4.0 m/s, on modern turbines, to 5.5 m/s, resulted in at least a 50% reduction in bat fatalities. This requires considerable cooperation on behalf of the project operators as curtailing turbine operations, even on a limited basis, clearly poses operational and economic restrictions resulting in some loss of revenue. This method does however offer a promising solution, particularly in areas where it has been proven that bat mortalities occur over a clearly defined and restricted time period. It is not yet clear whether this method of mitigation will prove sufficiently feasible and effective at reducing impacts to bats at costs that are acceptable to companies that operate wind energy facilities. Therefore, given the problems associated with the existing proposed methods of mitigation it is essential to investigate all other alternatives.

It has been suggested that the radio frequency (RF) radiation associated with radar installations could potentially exert an aversive behavioural response in foraging bats [21]. In 2006 Nicholls and Racey recorded bat activity along an electromagnetic gradient at ten radar installations throughout Scotland. Their results revealed that bat activity and foraging effort per unit time were significantly reduced in habitats exposed to an electromagnetic field (EMF) strength of greater than 2v/m when compared to matched sites registering EMF levels of zero. Even at sites with lower levels of EMF exposure (<2v/m), bat activity and foraging effort was significantly reduced in comparison to control sites.

Ahlén *et al*. [22] also reported anecdotal evidence that bats foraging offshore in Sweden avoided an area around Utgrunden lighthouse where a powerful radar was in permanent operation. However, although it has been demonstrated that large air traffic control and weather radars appear to exert an aversive response on foraging bats [21], this has little practical application in preventing bats from colliding with turbine blades. It is therefore necessary to establish whether a deterrent effect can be replicated with a small, portable radar system. It is also possible that the electromagnetic radiation from the radar may not be affecting bats directly but rather the insects upon which they feed. Bat activity within an area is strongly correlated with insect density [23], [24] therefore any reduction in insect density would result in a concurrent reduction in bat activity. In order to provide an efficient deterrent it is necessary to determine whether any observed reduction in bat activity is a direct result of exposure to electromagnetic radiation or an indirect result of a localised reduction in insect density.

Therefore the aims of the present study were to test the following hypotheses:

1. Bat activity will be reduced following exposure to a pulsed electromagnetic signal from a small portable radar unit.
2. The abundance of aerial insects will be reduced following exposure to a pulsed electromagnetic signal from a small portable radar unit.

## Materials and Methods

### Study sites and sampling protocol

In Britain, foraging bats are predominantly associated with areas where insect density is high: broadleaved woodland, particularly woodland edge, linear vegetation (tree lines and hedgerows) and riparian habitat. More open and intensively managed areas are avoided. In order to assess the impact of radar on foraging bats it was important to locate foraging sites with a high level of bat activity. Using existing knowledge obtained from detailed radio telemetry projects [25] in conjunction with extensive acoustic surveys, 20 foraging sites, with a high and consistent level of bat activity, were selected. All foraging sites were located within a 100 km radius of Aberdeen in northeast Scotland (latitude 57°23' N, longitude 02°45' W) and were separated by a minimum straight-line distance of >1 km to ensure independence. Twelve of these sites were located within riparian habitats (small ponds, rivers and streams) and the remainder along the edge of woodland where the radar signal would not be attenuated by any obstruction.

The radar used throughout the study was a Furuno FR - 7062 X-band marine radar (peak power 6 kW, beamwidth: horizontal −1.9°, vertical −22°, rotation 24 rpm or 48 rpm) with a slotted waveguide array antenna (1.2 m) capable of transmitting at pulse lengths of 0.08 µs–0.8 µs depending on the range selected. At each site the radar antenna was placed on a platform 2 m above ground level, such that the core area of bat activity was directly in line with the radar beam. At each foraging site a control (no radar signal) and experimental trial (radar switched on) were carried out. Starting 45 minutes after sunset, bat activity was recorded for a period of 30 minutes during each trial and the order of trials were alternated between nights. To avoid pseudoreplication, recordings were carried out only once at each of the 20 sites.

As in most radar systems, the antenna of the radar usually swept through 360 degrees. For the current experiment this would reduce the extent of exposure along any radius. Therefore the experiment was repeated with the antenna of the radar fixed such that the radar signal was orientated directly towards the area of highest bat activity. Similarly the duration of exposure to the radar signal is dependent on the duty cycle of the radar transmitter (pulse length×pulse repetition frequency). Therefore the experiment was repeated at each site using two different pulse length/pulse repetition rates (0.08 *µ*s/2100 Hz, 0.3 *µ*s/1200 Hz,) with the radar antenna fixed to maximise exposure. A portable electromagnetic field meter (PMM 8053-Accelonix Ltd.) and isotropic field probe (EP-330 Isotropic E-Field probe-Accelonix Ltd.) were used to measure the maximum value (peak hold) of the electromagnetic field strength (EMF) of the radar in volts per metre (v/m) at three distances from the radar antenna (10, 20, 30 m) for each of the two radar settings implemented throughout the study.

### Bat activity recording

**Figure 1:**
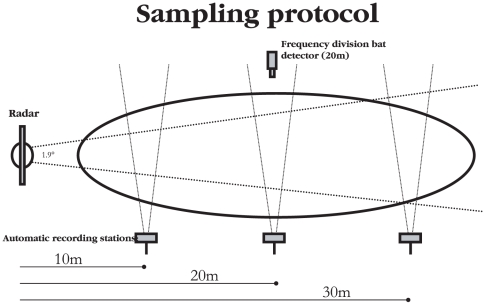
Sampling protocol of experimental trials carried out at 20 independent sites from July to September 2007. At each site bat activity was recorded for 1 h at three distances from the radar antenna (10,20,30 m) using three automatic bat recording stations orientated perpendicular to the radar beam. A frequency division bat detector was positioned 20 m from the radar antenna to provide further information on bat foraging activity during this period.

At each foraging site bat activity was recorded at three distances from the radar antenna (10, 20, 30 m) using automatic bat-recording stations [26]. Each automatic station consisted of a Batbox III heterodyne bat detector (Stag Electronics, Sussex, UK) linked to a count data logger (Gemini Data Loggers, UK Ltd, Chichester, UK) via an analogue to digital signal converter (Skye instruments, Ltd). The signal converter converts analogue signals from the bat detector into digital signals that can be recorded by the data logger. Every 0.5 seconds a positive or negative signal is sent to the data logger indicating the presence or absence of ultrasound respectively. Therefore the recorded number of bat active half seconds referred to as ‘bat counts’ over a thirty-minute trial provides a quantitative index of bat activity during that period. Most narrowband detectors will detect a range of frequencies centred on the value shown on the tuning dial. For the Batbox III this window is±8 kHz of the tuned frequency, therefore the frequency was set to 50 khz in order to effectively detect each of the 5 breeding species of bat in Scotland (*Pipistrellus pipistrellus*, *Pipistrellus pygmaeus*, *Myotis daubentonii*, *Myotis nattereri* and *Plecotus auritus*). The component parts of the system were housed in large plastic boxes with a hole cut for the bat detector microphones. Automatic recording stations were positioned on platforms 1.5 m above the ground and orientated perpendicular to the radar signal (Fig. 1).

In conjunction with the automatic recording stations bat activity was recorded continuously during each trial using a frequency division bat detector (S-25, Ultrasound Advice, London). This method of ultrasound transformation allows calls to be recorded in real time on audiocassettes and the number of recorded passes provides a quantitative assessment of bat activity Bat detectors were linked to a tape recorder (Sony Professional Walkman, Tokyo, WMD6C) containing metal-tape cassettes. At each site the bat detector was placed at a distance of 20 m from the radar antenna and the height and direction remained constant at 70 cm. The 60 minutes recording at each site were analysed using BatSound software (BatSound Pro, Pettersson Elektronic AB, Uppsala Sweden). In addition to the total number of bat passes, terminal feeding buzzes at each site were counted. These characteristic sounds are produced by aerial hunting and trawling vespertilionid bats when prey capture is attempted [27] and can be used to quantify foraging activity within a site. Foraging rate is expressed as the ratio of terminal buzzes to bat passes; this feeding buzz ratio (FBR) provides a measure of foraging intensity per unit of flight activity [28]. The use of frequency division detectors also allowed accurate species identification at each site.

### Insect Abundance

From July to September 2008 aerial insects were sampled using two identical Pirbright-Miniature light-suction traps (PMLT) [29] equipped with 8 W UV light bulbs. Each trap operated at 220 V transformed to 12 V to run from a car battery. At the base of each trap was a water-filled collecting vessel containing 2–3 drops of detergent. Most large insects were excluded by a large-mesh screen immediately above the fan and below the light bulb. The traps were deployed at 16 of the 20 foraging sites described above and were switched on for one hour prior to sunset. At each site the traps were positioned approximately 40 m apart with their trap inlets 2 m above ground level. On each sampling night the radar antenna was positioned on a platform 2 m above ground level and 10 m from one of the traps such that the antenna was orientated directly towards the trap inlet and fixed to produce a unidirectional signal. The second trap was positioned perpendicular to the radar beam to prevent any potential exposure to electromagnetic radiation and left to function as a control. To avoid any potential bias the selection of traps used as the control was alternated each night. The parameters of the radar tested were identical to those described above (Pulse length/pulse repetition rate: 0.08 *µ*s/2100 Hz; 0.3 *µ*s/1200 Hz,) no test was carried out with the antenna rotating.

Immediately following sampling, the insect catch was transferred from the collecting column into a 70% ethanol in water solution using a fine brush. Insects were then counted using a dissecting microscope (×30). Any insects with wingspans exceeding 20 mm were removed from the catch, as they would exceed the range of insect sizes captured by the species recorded throughout the study [30]. Following counting and sorting, the dry mass of insects was recorded by drying the samples in an oven until a constant mass was achieved (21 h).

### Statistical analysis

Differences in bat activity (bat counts and bat passes), bat foraging activity (feeding buzz ratios) and insect abundance between experimental and control trials were analysed using paired t tests. To account for multiple comparisons in paired t tests, we applied a manual Bonferroni correction (*P*-values×number of comparisons). However since the application of the Bonferroni correction increases the risk of making more type II errors, i.e. not recognising a true effect as significant [31] we report both corrected *P*_Bonferroni_ and uncorrected *P*-values. The effect of distance from the radar antenna was analysed using one-way ANOVA. Analyses were carried out using Minitab version 14 [32].

### Ethics statement

The authors' work on bats is licensed by the statutory nature conservation organisation in Scotland (Scottish Natural Heritage).

## Results

### Bat activity

Experimental trials were carried out during 58 nights from July 2007 till September 2007 representing a total of 58 hours of recording data within the following parameters:

1. Rotating antenna – pulse length/pulse repetition rate (0.08 *µ*s/2100 Hz) – 20 h
2. Fixed antenna – pulse length/pulse repetition rate (0.08 *µ*s/2100 Hz) – 20 h
3. Fixed antenna – pulse length/pulse repetition rate (0.3 *µ*s/1200 Hz) – 18 h

The maximum value (peak hold) of the electromagnetic field strength within these parameters is shown in Table 1. Field strength diminished slightly with increasing distance from the antenna under all radar parameters. However when the radar antenna was fixed to emit a unidirectional signal a fourfold increase in field strength was observed at all distances (Table 1).

**Table 1:** The maximum value (peak hold) of the electromagnetic field strength (v/m) at three distances from the radar antenna.

Antenna position	Pulse length (µs)	Pulse Repetition rate (Hz)	Duty Cycle (%)	EMF (v/m) Peak hold (10 m)	EMF (v/m) Peak hold (20 m)	EMF (v/m) Peak hold (30 m)
Rotating	0.08	2100	0.0168	5.58	5.11	3.79
Fixed	0.08	2100	0.0168	26.24	22.99	20.25
Fixed	0.3	1200	0.036	25.52	18.68	17.67

The three automatic stations recorded a total of 102,810 bat counts during 58 h of recording (Table 2). No significant difference was observed in the number of bat counts recorded between automatic stations positioned at 10, 20 and 30 m from the radar antenna (ANOVA, rotating antenna with pulse length 0.08 *µ*s: *P* = 0.57; fixed antenna with pulse length 0.08 *µ*s: *P* = 0.64; fixed antenna with pulse length 0.3 *µ*s *P* = 0.68) therefore all further tests were carried out on the average of these three values. A further 53,731 bat passes were recorded with the frequency division detector (Table 2). As expected, the majority of passes (84%) were attributed to the two cryptic pipistrelle species: *Pipistrellus pygmaeus* and *P. pipistrellus* (51% and 33% respectively) which are the most common and abundant bats in Scotland. A further 16% of bat passes were attributed to *Myotis daubentonii*.

**Table 2:** Total numbers of bat counts, bat passes and feeding buzzes recorded within treatment and control trials during 58 h of recording.

Index of bat activity	Rotating antenna (0.08 µs/2100 Hz)		Fixed antenna (0.08 µs/2100 Hz)		Fixed antenna (0.3 µs/1200 Hz)	
	Treatment	Control	Treatment	Control	Treatment	Control
Bat passes	11160	11599	8065	9305	5367	8235
Feeding buzzes	3711	4015	2386	3300	1563	2720
Bat counts (10 m)	6052	6275	4998	5974	3241	5517
Bat counts (20 m)	6364	6820	5261	6183	3494	5525
Bat counts (30 m)	7066	7386	5744	6792	3879	6239

**Figure 2:**
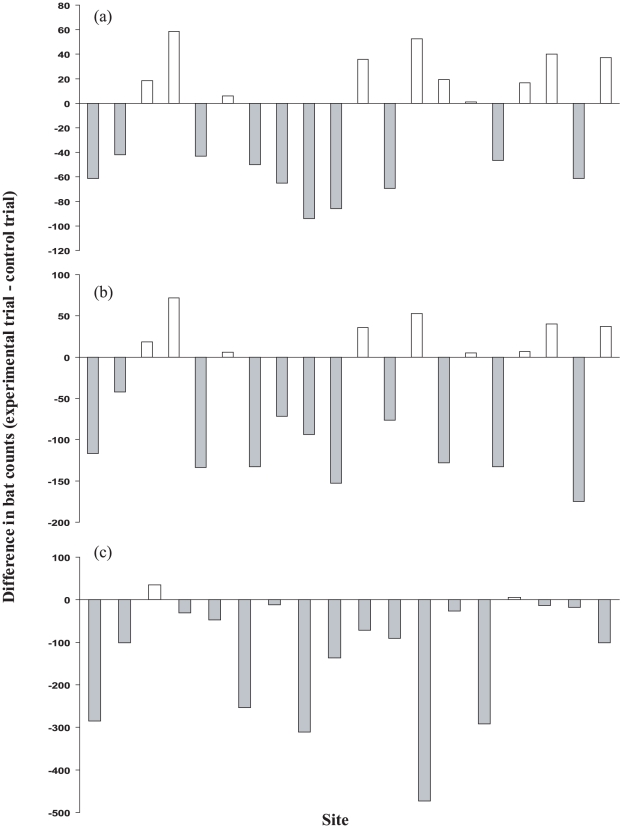
The response of bats to: (a) short pulse length (0.08 *µ*s) radar signal from a rotating antenna. (b) short pulse length (0.08 *µ*s) signal from a fixed antenna. (c) medium pulse length (0.3 *µ*s) signal from a fixed antenna. Each bar represents the difference in bat counts (the number of times that ultrasound was detected by the automatic bat recording stations) between control and experimental trials. A negative value indicates that bat activity was higher during the control trial than during the experimental trial when the radar was switched on.

**Figure 3:**
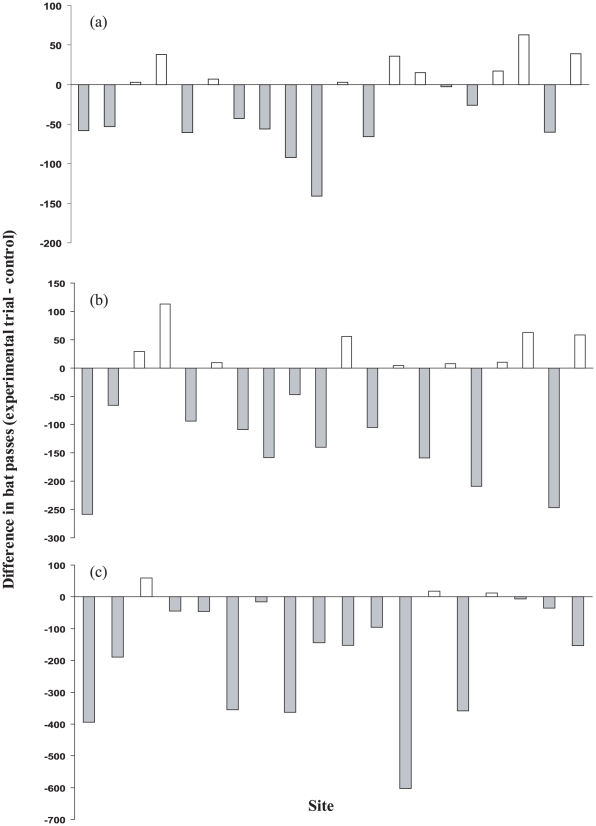
The response of bats to: (a) short pulse length (0.08 *µ*s) radar signal from a rotating antenna. (b) short pulse length (0.08 *µ*s) signal from a fixed antenna. (c) medium pulse length (0.3 *µ*s) signal from a fixed antenna. Each bar represents the difference in bat passes (recorded using a frequency division bat detector) between control and experimental trials. A negative value indicates that bat activity was higher during the control trial than during the experimental trial when the radar was switched on.

**Figure 4:**
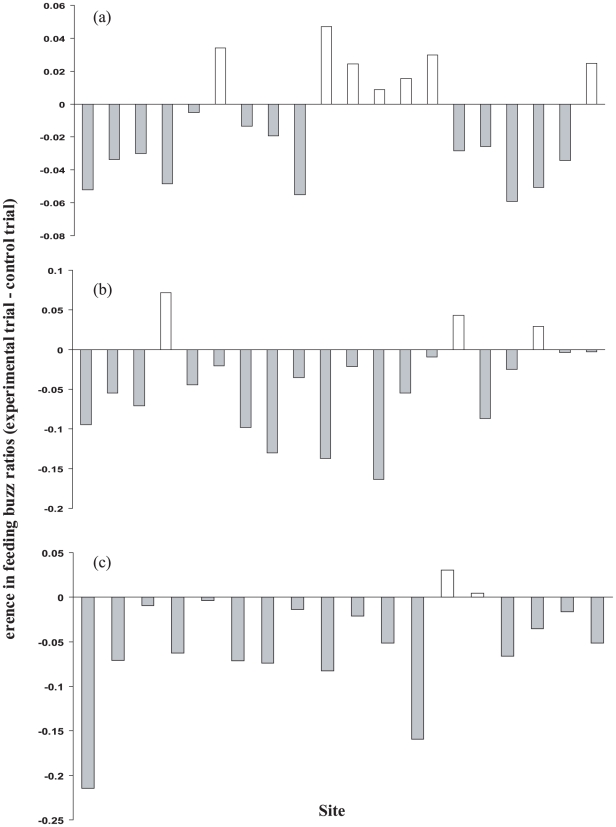
The response of bats to: (a) short pulse length (0.08 *µ*s) radar signal from a rotating antenna. (b) short pulse length (0.08 *µ*s) signal from a fixed antenna. (c) medium pulse length (0.3 *µ*s) signal from a fixed antenna. Each bar represents the difference in foraging rate per unit time as reflected by the difference in feeding buzz ratios (FBR) between control and experimental trials. A negative value indicates that bat activity was higher during the control trial than during the experimental trial when the radar was switched on.

Total bat activity was invariably higher during the control trials when compared to experimental trials (Table 2). However paired t tests carried out on all indices of bat activity (bat counts, bat passes, feeding buzz ratios) revealed no significant difference in bat activity between control and experimental trials when exposed to a short pulse length (0.08 *µ*s) radar signal from a rotating antenna (bat counts: t = 1.50; *P* = 0.151; *P*_Bonferroni_ = 0.453; Fig. 2a. Bat passes: t = 1.89; *P* = 0.074; *P*_Bonferroni_ = 0.222; Fig. 3a. FBR: t = 1.80; *P* = 0.088; *P*_Bonferroni_ = 0.264; Fig. 4a). Paired t tests carried out on all indices of bat activity (bat counts, bat passes, feeding buzz ratios) showed that bats were significantly less active during experimental trials than during control trials when exposed to a short pulse length (0.08 *µ*s) radar signal from a fixed antenna (bat counts: t = 2.87; *P* = 0.010; *P*_Bonferroni_ = 0.030; Fig. 2b. Bat passes: t = 2.54; *P* = 0.020; *P*_Bonferroni_ = 0.060; Fig. 3b. FBR: t = 3.82; *P* = 0.001; *P*_Bonferroni_  = 0.003; Fig. 4b). However, following Bonferroni correction the difference in the number of bat passes between experimental and control trials was no longer significant. Bats were also significantly less active during experimental trials than during control trials when exposed to a medium pulse length (0.3 *µ*s) radar signal from a fixed antenna (bat counts: t = 3.95; *P* = 0.001; *P*_Bonferroni_ = 0.003; Fig. 2c. Bat passes: t = 3.69; *P* = 0.002; *P*_Bonferroni_  = 0.006; Fig. 3c. FBR: t = 6.78; *P*<0.001; *P*_Bonferroni_  = 0.003; Fig. 4c). A summary of these results is presented in Table 3.

**Table 3:** Statistical significance of differences in bat activity between control and experimental trials (*) denotes a significant result for both corrected *P*_Bonferroni_ (*P* values×number of comparisons) and uncorrected *P*-values.

Antenna position	Index of activity	Pulse length (µs)	Pulse repetition rate (Hz)	Duty cycle (%)	n	t	P	P_Bonferroni_
Rotating	Bat passes	0.08	2100	0.0168	20	1.89	0.07	0.22
	Bat counts	0.08	2100	0.0168	20	1.50	0.15	0.45
	FBR	0.08	2100	0.0168	20	1.80	0.08	0.26
Fixed	Bat passes	0.08	2100	0.0168	20	2.54	0.02*	0.06
	Bat counts	0.08	2100	0.0168	20	2.87	0.01*	0.03*
	FBR	0.08	2100	0.0168	20	3.82	0.001*	0.003*
Fixed	Bat passes	0.3	1200	0.036	18	3.69	0.002*	0.006*
	Bat counts	0.3	1200	0.036	18	3.95	0.001*	0.003*
	FBR	0.3	1200	0.036	18	6.78	<0.001*	0.003*

### Insect Abundance

Experimental trials were carried out during 32 nights from July 2008 till September 2008 representing a total of 32 hours of recording data within the following parameters:

1. Fixed antenna – pulse length/pulse repetition rate (0.08 *µ*s/2100 Hz) – 16 h
2. Fixed antenna – pulse length/pulse repetition rate (0.3 *µ*s/1200 Hz) – 16 h

A total of 10 430 insects were caught during 32 hours of sampling per trap. Ninety five percent of the insects caught had wingspans <20 mm and were dried and included in further analyses. Paired t tests revealed no significant difference in insect abundance between control and experimental traps when exposed to either a short (0.08 *µ*s) or medium pulse length (0.3 *µ*s) radar signal (short pulse: n = 16, t = 1.50; *P* = 0.151; *P*_Bonferroni_ = 0.453; long pulse: n = 16, t = 1.89; *P* = 0.074; *P*_Bonferroni_ = 0.222).

## Discussion

Currently there is no accepted method of successfully mitigating bat collisions with wind turbines and attempts at deterring bats by the use of ultrasound have, as yet, been unsuccessful. Therefore the identification of alternative methods capable of inducing an aversive response in bats approaching turbine blades is of paramount importance. Very few field experiments have been carried out to ascertain the possible effects of high frequency electromagnetic radiation on populations of wild animals. However studies have shown that electromagnetic radiation can influence the development, reproduction, and physiology of insects [33], mammals [34], and birds [35]. Our results demonstrate that an electromagnetic signal from a small radar unit with a fixed antenna invariably reduced the foraging activity of bats within 30 m of the unit. However no significant decrease in activity was observed when the radar antenna was rotating. This is not surprising; the length of time a bat would be exposed to the radar signal is a function of the duty cycle of the radar signal (pulse length×pulse repetition rate) and the dwell time (the duration of time that a target remains in the radar beam during each rotation). The rotation of the radar antenna would reduce the time that bats were exposed to pulse-modulated microwave radiation and would therefore attenuate any potential deterrent effect. When the radar antenna was fixed to emit a unidirectional signal a fourfold increase in field strength was observed at all distances.

When foraging sites were exposed to a short pulse length signal from a fixed antenna there was a significant reduction in bat activity during experimental trials (bat counts and bat passes dropped by 15.5% and 13.3% respectively). Although once the Bonferroni correction had been applied, the difference in bat passes between control and experimental trials was no longer significant. An even greater level of significance was however observed when foraging sites were exposed to a medium pulse length signal from a fixed antenna (bat counts and bat passes dropped by 38.6% and 30.8% respectively). Clearly this represents a substantial reduction in bat activity. However, bats continued to forage at each site during experimental trials, and on no occasion were bats observed behaving abnormally or actively avoiding the beam of the radar. However, temporal and spatial fluctuations in bat foraging behaviour are common [23.24] and therefore results have to be treated with caution. Despite this caveat the significant reduction in bat activity during all experimental trials with a fixed antenna supports our hypothesis that electromagnetic radiation exerts a deterrent effect on foraging bats. This raises questions regarding the mechanisms through which bats could perceive electromagnetic fields and why they would seek to avoid them.

Nicholls and Racey [21] suggest that the aversive behavioural response of foraging bats to electromagnetic radiation may be a result of thermal induction. Studies investigating the behavioural response of laboratory animals to the presence of electromagnetic fields have shown that even short-term exposure can produce a thermal burden in an organism that can result in significant behavioural and physiological changes, some of which may be harmful [36]. Behavioural effects of such exposure include perception, aversion, work perturbation, work stoppage and convulsions [37]. The wing membranes of bats present a large surface area over which radiation might be absorbed, increasing heat load on the animal. This, combined with the heat energy produced during flight, makes bats particularly susceptible to overheating [38], [39]. Furthermore, observations of captive bats have noted their aversion to even a moderate infra-red heat source [40].

However the pulsed microwave radiation characteristic of radars is a rather inefficient source of energy. The energy produced by a radar signal can reach very high values of peak power density, at relatively low levels of power density averaged over time. This is because the pulse length of the radar signal is hundreds of times shorter than the pulse repetition rate, therefore the average value of power density is hundreds of times lower than the peak value of the radiation. Therefore it would seem unlikely that the energy in the radar signal would be sufficient to induce a thermal burden in bats foraging within the beam. However several studies have reported significant behavioural and physiological effects resulting from exposure to pulsed microwave radiation even when the average power density of the signal was relatively low [41]–[43]. The mechanism through which pulsed microwave radiation could affect behaviour in this manner is unclear although one possibility is an auditory response commonly referred to as the auditory microwave hypothesis.

The auditory perception of pulsed microwaves is now widely accepted. The effect is generally attributed to the thermoelastic expansion of brain tissue following the small but rapid increase in temperature due to the absorption of the incident energy. This generates a sound wave in the head that subsequently stimulates the cochlea. Repeated or prolonged exposure to these auditory effects is considered stressful [44].

Laboratory experiments have shown that the frequency of the induced sound is a function of head size and of the acoustic properties of the brain tissue. The estimated fundamental frequency of vibration in guinea pigs, cats and adult humans are 45, 38, and 13 kHz respectively [45], [46]. It is therefore not only plausible but probable that bats exposed to an RF pulse of sufficient power would effectively hear this pulse and the frequency detected would lie within the range of frequencies used for orientation, prey detection and capture for the majority of bat species. It is possible that, as reported in other studies, exposure to these auditory effects may be stressful for bats or indeed it may interfere with their echolocation, inhibiting prey detection or capture. During the present study, foraging rate per unit time was significantly reduced during experimental trials indicating that bats foraging within the exposed area were feeding at a reduced rate in comparison to those foraging during the control trials. This is particularly surprising given that exposure to the radar had no significant impact on the abundance of aerial insects, and the observed reduction in foraging rate is therefore unlikely to be linked to a decline in insect abundance. It is therefore possible that the auditory perception of the radar signal during experimental trials could have interfered with the bats ability to detect or capture prey. However further experimentation would be required to accurately identify the causal relationship between exposure to electromagnetic radiation and the observed reduction in both bat activity and foraging rate.

Although we have demonstrated a clear biological effect, one of the limitations of the present study was the use of a commercial marine radar that was not specifically designed for the task. With only a limited control over the parameters of the radar signal, it is difficult to determine which parameters are most effective in deterring bats. To better understand the response of bats to electromagnetic radiation, and to identify an optimum signal capable of deterring bats, will require radar engineers to work with bat biologists to develop a portable radar which can be manipulated to produce a wider range of electromagnetic outputs. The parameters most likely to be important are the frequency, pulse length/pulse repetition rate and power output of the signal. Similarly, the radar used in the present study was only effective when the antenna was fixed to produce a unidirectional signal with a horizontal beamwidth of 1.9°. A narrow unidirectional signal is clearly not appropriate to deter bats from approaching wind turbines. In order to provide an effective deterrent it would be necessary to emit a multidirectional electromagnetic signal capable of encapsulating the large volume of the rotor-swept zone.

### Conclusion

We have demonstrated that pulsed electromagnetic radiation from a small, affordable and portable radar system can reduce bat activity within a given area. Results were most effective when the radar antenna was fixed to produce a unidirectional signal therefore maximising dwell time within the beam of the radar. However although bat activity was significantly reduced during experimental trials substantial numbers of bats continued to forage within the beam. It is possible that only a particular combination of wavelength, pulse repetition rate, power output and target size or orientation may provoke a reaction and further work is necessary to elucidate this relationship further.
